# *“If they had a place to live, they would be taking medication*”: a qualitative study identifying strategies for engaging street‐connected young people in the HIV prevention‐care continuum in Kenya

**DOI:** 10.1002/jia2.26023

**Published:** 2023-06-02

**Authors:** Lonnie Embleton, Pooja Shah, Edith Apondi, David Ayuku, Paula Braitstein

**Affiliations:** ^1^ Centre for Global Health Dalla Lana School of Public Health University of Toronto Toronto Ontario Canada; ^2^ London School of Hygiene & Tropical Medicine London UK; ^3^ Moi Teaching and Referral Hospital Eldoret Kenya; ^4^ Department of Mental Health and Behavioural Science College of Health Sciences Moi University Eldoret Kenya; ^5^ Department of Epidemiology Dalla Lana School of Public Health University of Toronto Toronto Ontario Canada; ^6^ Academic Model Providing Access to Healthcare (AMPATH) Eldoret Kenya; ^7^ School of Public Health, College of Health Sciences Moi University Eldoret Kenya

**Keywords:** adolescents, HIV care continuum, Africa, key and vulnerable populations, structural drivers, adolescent girls and young women

## Abstract

**Introduction:**

Street‐connected young people (SCY) experience structural and social barriers to engaging in the HIV prevention‐care continuum. We sought to elicit recommendations for interventions that may improve SCY's engagement along the HIV prevention‐care continuum from healthcare providers, policymakers, community members and SCY in Kenya.

**Methods:**

This qualitative study was conducted in Uasin Gishu, Trans Nzoia, Bungoma, Nakuru and Kitale counties in Kenya between May 2017 and September 2018 to explore and describe the public perceptions of, and proposed and existing responses to, the phenomenon of SCY. This secondary analysis focuses on a subset of data interviews that investigated SCY's healthcare needs in relation to HIV prevention and care. We conducted 41 in‐depth interviews and seven focus group discussions with 100 participants, of which 43 were SCY. In total, 48 participants were women and 52 men.

**Results:**

Our analysis resulted in four major themes corresponding to stages in the HIV prevention‐care continuum for key populations. We identified the need for an array of strategies to engage SCY in HIV prevention and testing services that are patient‐centred and responsive to the diversity of their circumstances. The use of pre‐exposure prophylaxis was a biomedical prevention strategy that SCY and healthcare providers alike stressed the need to raise awareness around and access to for SCY. Several healthcare providers suggested peer‐based approaches for engaging SCY throughout the continuum. However, SCY heavily debated the appropriateness of using peer‐based methods. Structural interventions, such as the provision of food and housing, were suggested as strategies to improve antiretroviral therapy adherence.

**Conclusions:**

This study identified contextually relevant interventions that should be adapted and piloted for use with SCY. Education and sensitization of SCY and healthcare providers alike were identified as possible strategies, along with affordable housing and anti‐poverty strategies as cash transfers and provision of food. Peer‐based interventions are a clear option but require SCY‐specific adaptation to be implemented effectively.

## 1 INTRODUCTION

Young people aged 10–24 years, particularly adolescent girls and young women (AGYW) and other young key populations (YKPs), are still disproportionately acquiring HIV in sub‐Saharan Africa (SSA) [1]. In many SSA countries, low proportions of young people have ever been tested for HIV and estimates suggest only 60% of young people living with HIV are aware of their HIV status [2, 3]. Despite the increased availability of antiretroviral therapy (ART), many young people do not initiate ART (∼51%), and for those who do, few achieve viral suppression [2, 3, 4, 5, 6, 7, 8]. Across the HIV prevention‐care continuum, young people face substantial barriers to engaging and re‐engaging in care. These barriers may include, but are not limited to, poverty, stigma, gender inequality, restrictive laws requiring parental/guardian consent to access HIV services and poor quality adolescent care [2, 9, 10, 11].

YKPs are categorized as young men who have sex with men, transgender youth, young people who inject drugs, young people who sell sex and young people involved in the criminal justice system (e.g. those who are incarcerated, experience discrimination/harassment by police, etc.) [12, 13, 14, 15, 16, 17], have been identified as young people most vulnerable to acquiring HIV [12, 14]. YKPs face additional challenges to engaging in care due to laws that criminalize sex work, drug use and same‐sex relations, and being subject to violence and severe stigma and discrimination due to their social identities [18, 19]. Young people who find themselves living and/or working on the streets in SSA, termed street‐connected young people (SCY), are a population at high risk of acquiring HIV [20, 21, 22, 23, 24]. SCY are frequently overlooked and not explicitly categorized or defined as a YKP in the literature [12, 16], but often fall within one or more of the YKP groups, although SCY may not self‐identify with any of the existing YKP categories.

SCY, for whom the streets play a central role in their everyday lives and social identities [25], have a highly stigmatized identity that influences their ability to access and engage with the health system [26, 27]. Many SCY in SSA may be inadequately or precariously housed or experience absolute homelessness [20, 28, 29], which has been identified as an independent risk factor for HIV acquisition and disease progression [30]. Moreover, SCY frequently experience profound levels of violence [31, 32], including from authorities, and are often involved in the criminal justice system [29, 33, 34, 35]. Finally, many SCY, particularly AGYW connected to the street, engage in transactional and commercial sex work that elevates their vulnerability to acquiring HIV [23, 36, 37, 38, 39, 40].

In Kenya, SCY are numerous [20, 21, 41, 42, 43] and have an HIV prevalence between 4.1% and 8.1% [20, 21, 22, 23, 24], exceeding that of other young people aged 15–24 in the country, which is estimated to be 1.98% [44]. Street‐connected young women may be disproportionately acquiring HIV, with 10.8% of those aged 15–24 years, and 26.8% of those aged 25–29 years testing HIV positive in Eldoret, Kenya [20]. SCY living with HIV may not be accessing HIV treatment services, as evidence has demonstrated that 37% of deaths among SCY in this town were attributed to HIV/AIDS; 59% among deceased street‐connected young women and 26% among street‐connected young men [45, 46].

Despite being an HIV endemic region and a population with multiple vulnerabilities, there has been very limited research on interventions tailored specifically for SCY to improve engagement in the HIV prevention‐care continuum [24, 47, 48, 49, 50, 51]. To fill this gap and identify contextually relevant strategies to increase SCY's engagement in the HIV prevention‐care continuum, we sought to elicit recommendations for interventions and services from healthcare providers, policymakers, community members and SCY in Kenya.

## 2 METHODS

### 2.1 Study design

From May 2017 to September 2018, we conducted a qualitative study, informed by Bronfenbrenner's socio‐ecological theoretical framework [24], which sought to investigate community perceptions of, and proposed and existing solutions to, the occurrence of SCY. This secondary analysis uses a subset of data from focus group discussions (FGDs) and in‐depth interviews (IDIs) that investigated SCY's healthcare needs, including those related to HIV.

### 2.2 Study setting

This study occurred in Kitale, Bungoma, Kisumu, Nakuru and Eldoret, Kenya. These towns were purposively selected due to the number of SCY who are known to reside in these locations [20, 21, 41, 42, 43]. Eldoret, the administrative capital of Uasin Gishu (UG) County and the location where the study investigators are based, was the principal study site. Eldoret is home to Moi University, Moi Teaching and Referral Hospital (MTRH) and the Academic Model Providing Access to Healthcare (AMPATH), a long‐standing partnership between Moi University, MTRH and a consortium of universities from North America [52].

### 2.3 Study participants

This study sought to enrol 100 participants who had knowledge of and experience interacting with SCY to gain a diverse range of perspectives on the issue of SCY. In Eldoret, we engaged the County Children's Coordinator, Children's Officers, police officers, community leaders (Chiefs and Elders), vendors, general community members, stakeholders, parents of SCY, former and current SCY, peer navigators [24] and healthcare providers at MTRH and AMPATH. The research team's long‐standing relationship with the local community in UG County facilitated reaching this diverse group of participants. Across Kisumu, Kitale, Nakuru and Bungoma, we engaged Children's Officers, police officers and SCY.

### 2.4 Sampling, recruitment and enrolment

All participants in this study were purposively sampled, however; we used different recruitment strategies across categories of participants. A gender and an age‐balanced purposive sample of SCY aged 15–24 years in all counties were recruited from locations where they are known to live, work and play. In each of these locations, study sensitization and street outreach occurred, where the purpose of the study was explained and SCY were invited to voluntarily participate. In UG, SCY were invited to the Rafiki Center of Excellence for Adolescent Health at MTRH to undergo enrolment and consent. In other counties, SCY were enrolled and gave consent in street venues.

For government officials (e.g. County Children's Coordinator and Children's Officers), we invited those directly responsible for child protection activities in each county to participate through a formal letter that explained the purpose of the study and followed up in person. Government officials were consented and interviewed in their offices. Community leaders, vendors, police officers, general community residents, parents of SCY and stakeholders were purposively sampled through existing relationships and networks and contacted in person or by phone to explain the purpose of the study and invite them to voluntarily participate. Healthcare providers, including social workers, clinical officers, nurses, peer navigators, and HIV testing and counselling practitioners from MTRH and AMPATH were purposively sampled and contacted through our established networks. In UG county, participants were invited to MTRH or Moi University offices to undergo informed consent and participate in interviews. In other counties, participants were consented and interviewed in their offices and places of employment.

### 2.5 Ethical considerations

This study received ethics approval from the MTRH Institutional Research Ethics Committee and the University of Toronto Research Ethics Board. Written informed consent was obtained from all participants. The study received a waiver of parental consent for minors and followed an established process for conducting ethical research with SCY [53]. SCY were asked to provide documented verbal assent (those aged 15–17 years) or consent (those aged 18–24 years) to participate. All participants were made aware that their interviews would be audio‐recorded; nine participants (police officers and Children's Officers) declined to be audio‐recorded but agreed to be interviewed and gave the interviewer permission to take notes. Government officials were compensated for their time with 1000 Ksh (∼ $US10.00), and community participants and SCY with 200 Ksh (∼US$2.00).

### 2.6 Data generation

We used two separate interview guides designed and pre‐tested by a local team of investigators: one for SCY and one for other participants. In the first guide, SCY were asked broadly about their experiences and interactions with the communities where they live and work. As well, SCY were asked about what should be done to assist SCY and about their experiences accessing healthcare. A subset of questions related to healthcare needs focused on HIV as an issue in the street community and asked participants about HIV prevention strategies, including the use of peer educators, community education and life skills. The second guide asked participants about their general perceptions of SCY, their experiences interacting with SCY, perceptions of their needs, and their thoughts on SCY accessing healthcare, how they are treated, and what kind of healthcare services and care are important and feasible for the population. For healthcare providers, additional questions in relation to the provision of healthcare to SCY were included. A subset of questions focused specifically on HIV testing and counselling and HIV prevention strategies. Data were triangulated from multiple sources (e.g. interviews with different types of participants in different locations) to enhance the reliability and validity of the data and reach saturation [54].

A team of eight trained interviewers conducted seven FGDs and 41 IDIs with 100 participants: 48 women and 52 men (Table 1). In total, 22 interviews were conducted in English, and 26 were conducted in either Swahili or a mix of Swahili and English. For SCY, interviewers incorporated *Sheng* (dialect used on the street). Typically, FGDs took one and a half hours and IDIs 40 minutes.

**Table 1:** Participants, interviews and location

Social actors	# of interviews	Location	Gender of interviewees
Community leaders	4	Uasin Gishu	4 men
County Children's Coordinators	1	Uasin Gishu	1 man
Police officers	6	Uasin Gishu, Nakuru Trans‐Nzoia, Kisumu, Bungoma	3 women and 3 men
Children's Officer(s)	6	Uasin Gishu, Nakuru Trans‐Nzoia, Kisumu, Bungoma	2 women and 4 men
Vendors	2	Uasin Gishu	1 woman and 1 man
General community	3	Uasin Gishu	1 woman and 2 men
CBO/stakeholders and SCY advocates	6	Uasin Gishu	2 women and 4 men
Peer navigators	2	Uasin Gishu	1 woman and 1 man
Parents of street children	1	Uasin Gishu	1 woman
Former street‐connected youth	3	Uasin Gishu	2 women and 1 man
Street‐connected youth	7	Uasin Gishu, Kisumu, Trans‐Nzoia	5 women and 2 men
Total in‐depth interviews	41		18 women and 23 men
AMPATH clinicians	FGD	Uasin Gishu	2 women and 3 men
AMPATH nurses, social work, counsellors	FGD	Uasin Gishu	4 women and 2 men
MTRH clinicians	FGD	Uasin Gishu	6 men
MTRH nurses	FGD	Uasin Gishu	6 women
SCY males FGD	FGD	Uasin Gishu	12 men
SCY females FGD	FGD	Uasin Gishu	12 women
Mixed gender SCY Nakuru	FGD	Nakuru	6 young women and 6 young men
Total number of FGDs	7		30 women and 29 men

### 2.7 Qualitative data analysis

We conducted a thematic analysis [55]. Our analytic process commenced with an in‐depth reading of our data. Next, we generated an initial set of codes related to healthcare and a sub‐set of codes on HIV, including identifying HIV as an issue for SCY, HIV prevention, testing and counselling, education, disclosure, provision of HIV services, availability of HIV services, and HIV care and treatment. The codebook was finalized by repeatedly testing its validity and comprehensiveness through test‐coding transcripts. Transcripts were coded by four investigators and compared for consistency. In a series of interpretive meetings, we discussed analytic notes and further defined and refined themes. For this secondary analysis, we situated our HIV‐related findings into major themes associated with the HIV prevention‐care continuum for key populations [19].

## 3 RESULTS

**Figure 1:**
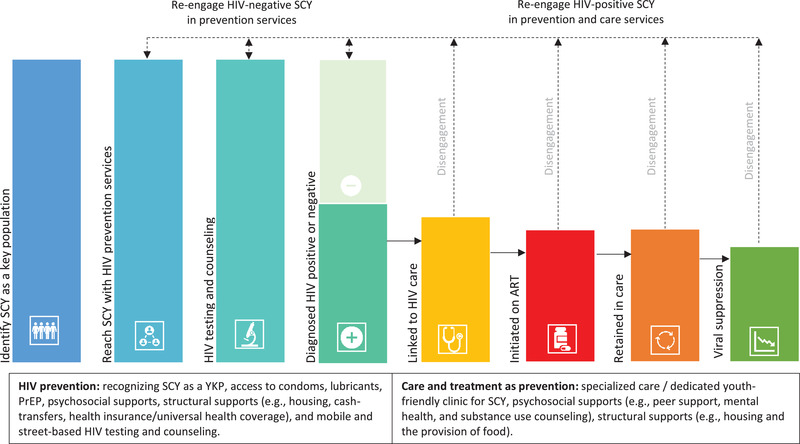
HIV‐prevention care continuum for SCY as a YKP.

The study included 100 participants, of which 43 were SCY: 23 street‐connected young women and 20 street‐connected young men. The median age of SCY was 16 years and 42 years for other participants interviewed. Our analysis resulted in four major themes that correspond to stages in the HIV prevention‐care continuum for key populations: (1) identifying SCY as a YKP; (2) reaching SCY with HIV prevention services; (3) HIV testing and counselling; (4) initiating care and adherence to ART. Figure 1 presents an adapted HIV‐prevention care continuum for SCY as a YKP [19].

### 3.1 Identifying SCY as a young key population

SCY consistently and unanimously recognized that HIV was a significant issue for their community, as evidenced by a group of street‐connected young men in Eldoret who stated in unison “*it is a problem*.” One former street‐connected young woman affirmed this by describing the impact HIV has had on the population of girls on the street in Eldoret: “*The girls are many but most of them have died due to HIV. Many of the girls I started with [on the street] are dead*.”

Young women connected to the streets described at length their heightened vulnerability to acquiring HIV for numerous reasons associated with their street involvement. Young men's engagement in multiple concurrent partnerships leaves them highly susceptible to acquiring HIV they explained:

You may sleep with someone whom you don't know is infected…. It is a problem because your boyfriend might sleep with your friend who is infected then he sleeps with you too….Many girls are suffering because of sharing these boys, they will sleep with you and the next day you see him with your friend.

(Eldoret, FGD, street‐connected young women)

Young women also discussed their inability to negotiate condom use with young men under the influence of alcohol and other substances, and explained how their own substance use leaves them vulnerable to sexual and gender‐based violence (SGBV):

You may find someone who will tell you that he doesn't want to use a condom because it is bad, or he has sniffed gum [glue] and taken alcohol, so he is confused. The small girls sniff glue and it becomes too much for them, then the boys come to them and force themselves on them.

(Eldoret, FGD, street‐connected young women)

Young men confirmed perpetrating SGBV and recognized the risk of acquiring or transmitting HIV when doing so:

Even if I seduce those girls, I don't have money or a house, so I sleep outside. So, I will just buy her cheap alcohol and then take her to Mangula [barracks location] because there I won't have to pay for anything. Then, when they [other street boys] see I have taken her to the forest they will follow me, and around 30–40 will also have sex with her. So, if she is sick, we will all be infected. But for those who are clever like me, we won't share.

(Eldoret, FGD, street‐connected young men)

SCY explained that experiencing SGBV was linked to other structural determinants of health inequities, including precarious and unsafe housing:

You see, everyone should be cautious, but in our set up [housing] is difficult. You see this room, we sleep many of us, and those that dont fit, sleep outside the door. At night the boy next to you just comes ontop of you and has sex with you. He then taps the other boys who in turn do the same. This continues until all the boys have had sex with you ‐ you can even get infected.

(Nakuru, FGD, SCY)

SCY's multiple and intersecting vulnerabilities make them a YKP highly susceptible to acquiring HIV, who require biomedical, behavioural and structural HIV prevention strategies that are tailored to their complex circumstances living and/or working on the streets.

### 3.2 Reaching SCY with HIV prevention services

Several biomedical and behavioural approaches for HIV prevention for SCY were recommended. The use of pre‐exposure prophylaxis (PrEP) was discussed by healthcare providers and SCY. As one street‐connected young man in Kisumu shared: “*We don't want our brothers and sisters to die as we watch, so we should teach them and most importantly give them PrEP*.” The need for combined behavioural and biomedical strategies was commonly suggested by healthcare providers. A group of clinical officers recommended that SCY should be made aware of and offered PrEP and said condoms were another biomedical approach readily available: “*We try using condoms, placing them in the toilets. Those that come to us personally, we pack for them nicely in an envelope. When you put them in the toilets they take everything. I think we should also have PrEP for them, they should know that that service is available.”*

A group of nurses brought up reducing HIV‐related stigma in the street subculture and dispelling myths and misconceptions about HIV transmission, as an important component of HIV prevention for SCY:

Yes, it affects them because when they know this one is positive, they run away from that person and they don't know HIV is gotten through blood and other fluids. There was a time they came to our ward and the patient was positive so they used to ask if HIV can be treated completely. They fear talking to them and greeting them.

(FGD, Nurses)

Several healthcare providers recognized the importance of augmenting HIV literacy and knowledge in a culturally suitable manner, ensuring HIV prevention materials are tailored to SCY and use appropriate and comprehensible language. Further, the use of peer educators with experience being street‐involved was suggested by a group of healthcare providers to improve communication about HIV prevention:

We need to design materials that can be used by them, you find most of them are done in normal Swahili and English, yet they have their own language, even the person giving the talk talks to them like he is dealing with any other person. You can use someone who was on the streets and got rehabilitated.

(FGD, Nurses, Counsellors, and Social Workers)

Some SCY concurred that talking to peers would be a welcome strategy to learn about HIV as explained by one young man in Kitale: “*Yes, I would, I would feel free to talk about HIV with my friends. I would like to learn the HIV messages, it will make me feel good to learn and go and teach others*.” However, the use of peers for HIV education was highly contested among SCY. Not all street‐connected participants agreed that peers who are their age mates are suitable to provide HIV prevention education as one group of street‐connected young women debated:

Interviewer (I): Would you listen to your age mate?

Respondent (R): I would.

R: Some of us are too arrogant to listen to our age mates.

I: Why is that so?

R: If I talk to this one, she will ask me what I am telling [her] and chase me away.

R: It is good if you find someone older.

R: If you call a group not everyone will listen to you.

R: If this one talks to me, I will feel like she is not telling me anything important because she is my age mate, an older person would be better.

R: If you tell her about HIV, she will think that you want her man so she will ignore you.

(Eldoret, FGD, street‐connected young women)

Several SCY across geographic locations felt that it would be better if a doctor or someone who is older and educated were to provide education about HIV prevention:

I: Who do you think should be talking to street youth about HIV prevention?

R: A doctor or someone like you, some one who has gone to school.

I: Do you feel more comfortable when your peers or someone closer to your age talk about HIV prevention?

R: No! No one will listen to them.

(Nakuru, FGD, SCY)

Although there was disagreement regarding the use of peers to deliver HIV prevention education, participants made clear that there are several gaps in HIV prevention programming for SCY and that a variety of HIV prevention strategies should be tailored to the diverse needs of SCY.

### 3.3 HIV testing and counselling

Unanimously, healthcare providers iterated the importance of SCY knowing their status and getting tested for HIV, as stated by a nurse: “*They need to know their status like we are all encouraged to get tested and those positive can get access to HIV care*.” Notwithstanding the debate among SCY regarding the role of peer educators, a clinical officer suggested that peers may be influential in promoting the uptake of HIV testing and counselling: “*You know they work as a unit, so if you can get peer friendly ambassadors within themselves, they can influence others to know their status and you can also use programs to teach these peers on how they can teach others*.”

Despite the widespread availability of free HIV counselling and testing in Kenya, one street‐connected young man discussed an inability to pay as a barrier to HIV, suggesting the need for SCY to be covered by national health insurance: “*Some of us usually go for testing but when we get there, they ask us to pay, yet we don't have money, so we just leave*.” However, most often, SCY discussed going to a clinic‐based setting to get tested without an issue: “*We just go to the clinic and get tested*.” Some participants discussed different strategies, such as mobile testing clinics, that may increase engagement in HIV testing and counselling, as one street‐connected young man in Kitale offered: “*It would help if they got mobile testing clinics here in the morning*.” However, a street‐connected young woman in Kitale countered this idea and felt that testing in locations where SCY live, work and play was inappropriate: “*They play football here. If won't be a good idea to learn about or get tested for HIV at half time, they don't want it in a public place*.” Overall, participants pointed to the need for an assortment of youth‐friendly options and strategies for SCY to select from to increase the uptake of HIV testing and counselling.

### 3.4 Initiating care and adherence to treatment

Once diagnosed, initiating care and adherence to ART were discussed as important issues by healthcare providers and community members alike. A clinical officer in Eldoret recommended integrating HIV care into youth‐friendly centres: “*We used to have centers called youth‐friendly centers, I don't know what happened to them, they were under some NGO. If we want to help these guys [SCY] we should come up with such centers, it was a center for them to integrate and they got HIV care also*.”

Several participants recognized that SCY may require their own clinic or specialized care where they receive ART. A female vendor in Eldoret suggested: “*those who are HIV‐positive should have their own place where they can go for ART, those with children should be sensitized to be taking their children for clinics and they will be okay*.”

Challenges with adherence to ART for SCY was brought up as a fundamental issue across participants. As one parent of SCY explained, homelessness is a crucial barrier to taking medication, and the provision of housing may improve adherence: “*they can be given medication, but they throw them away when they get to town. Some may be positive but they don't take medication, but if they had a place to live, they would be taking medication*.”

One stakeholder in Eldoret who works with SCY suggested that they needed a dedicated individual to follow up to ensure they can adhere to treatment: “*Some of them don't adhere to HIV treatment and they die due to HIV complications so there should be someone to check on them*.” Clinicians discussed that the use of substances, such as sniffing glue, was also a major challenge to adherence and treatment and suggested that SCY require counselling: “*If infected there is treatment, but they need proper education because the risk of non‐compliance is very high, once they sniff the gum [glue] they will forget what they are supposed to do, so they need proper counselling*.”

Access to food and adequate nutrition also arose as a significant barrier to taking drugs as one group of healthcare providers stated: “*when it comes to adherence of drugs they are always defaulting because they will only take the drugs if they eat*.” SCY in Kitale concurred, that those who are HIV positive should be allocated food: “*People should give us a daily ration from the district hospital, those who have HIV should get the food*.” The need for adequate nutrition to adhere to ART was also recognized by one Children's Officer:

They deserve it [specialized healthcare] and that is why we should be able to have an organized place where they can be reached and given these services otherwise you won't expect them to adhere to the drugs while on the streets. There should be a place to guide and feed them.

(Children's Officer)

## 4 DISCUSSION

This qualitative analysis identified several avenues to improve SCY's engagement in the HIV prevention‐care continuum. First, this work provides additional evidence to identify SCY as YKP who require tailored interventions and services to address their complex needs and barriers they encounter to engaging and re‐engaging in care. Second, this analysis identified the need for an array of strategies to engage SCY in HIV services that take a patient‐centred approach, as even among SCY, there was debate about the appropriateness of strategies such as the use of peers. Finally, our analysis highlighted the need to integrate structural interventions into the delivery of HIV services for SCY to address the impact of issues such as food insecurity and homelessness.

SCY in this study recognized their heightened vulnerability to HIV and explained how their circumstances, such as living in unsafe housing, substance use and SGBV in the street subculture, drive their increased risk. A body of evidence supports that SCY face multiple and intersecting vulnerabilities and inequities that elevate their risk of acquiring HIV, and that in the context of Kenya, SCY and particularly AGYW connected to the street, have a higher prevalence and incidence of HIV than non‐street connected young people [20, 22, 24, 38, 39, 56, 57, 58]. Moreover, a large proportion of SCY's deaths in this context have been attributed to HIV [45, 59]. Yet, SCY in SSA are generally overlooked as a separate category of individuals considered a YKP highly vulnerable to acquiring HIV. Key populations have been exclusively defined as men who have sex with men, people in prisons and other closed settings, people who inject drugs, sex workers and transgender people [12, 16, 18, 19, 60]. Although the World Health Organization recognizes that people from key populations may be homeless [60], individuals who identify as SCY are not recognized as a YKP. Undertaking population size estimates, mapping activities and HIV‐prevalence studies are a strategic component of programme planning and identifying key populations [19, 61]. Population size estimation, mapping and HIV‐prevalence studies should occur across urban SSA contexts with substantial populations of SCY to further estimate and characterize the burden of HIV in this highly marginalized group. One strategy to do so may be to use a point‐in‐time count approach integrating HIV testing and counselling [20]. Identifying SCY as a distinct YKP is an important component to prioritize funding for implementing and planning programmes to engage this highly marginalized population in the HIV prevention‐care continuum [61].

The current analysis points to the need for an array of tailored HIV prevention strategies for SCY. Several healthcare providers suggested peer‐based approaches to engaging SCY along the continuum. Though, SCY in this study heavily debated the appropriateness of using peer‐based approaches. Peer‐based strategies as a component of HIV prevention education have shown mixed effectiveness [62, 63, 64]. While in Kenya, peer navigators have been highly effective at engaging SCY in HIV testing and counselling [24]. However, given the mixed effectiveness of peer‐based approaches to HIV prevention education [63], and some SCY's averseness to engaging with peers, an over‐reliance on peer‐based strategies alone to improve SCY's engagement in the HIV prevention‐care continuum may not be advisable. Instead, peer‐based approaches may be one point in a multi‐pronged approach to engaging SCY in HIV services.

PrEP was a biomedical prevention strategy that SCY and healthcare providers alike stressed the need to raise awareness around and access to for SCY. Offering PrEP to groups of young people who are highly vulnerable to acquiring HIV is one of the five pillars to strengthen HIV prevention for adolescents and young people [65]. Given AGYW's reliance on transactional and survival sex in the street subculture [36, 38, 39, 56, 66], there is a pressing need to provide PrEP to SCY and conduct implementation science research to identify feasible, appropriate and cost‐effective approaches to improve the uptake and delivery of PrEP to SCY in this context.

Finally, while participants did not specifically mention any structural HIV prevention strategies to use with SCY, structural risk factors for HIV acquisition were discussed and structural interventions, such as housing and the provision of food as strategies to improve ART adherence, were suggested. SCY face several structural and social inequities, including a lack of stable housing, abject poverty, repressive laws that criminalize street involvement, violence, and poor nutrition and food insecurity, all of which may act as barriers to engaging in the HIV prevention‐care continuum in Kenya [33]. Addressing the structural factors that impact SCY's engagement in the HIV continuum will require multiple interventions and policies. Evidence supports that cash transfers and anti‐poverty programmes are an effective structural intervention to ameliorate HIV outcomes along the continuum [67]. In addition, early evidence suggests that some low‐cost strategies may be feasible and effective. For instance, the provision of a healthy nutritious meal in combination with peer navigation services integrated into the health system may be an avenue to improve ART adherence and viral suppression among SCY living with HIV [51]. Further, the lack of safe and adequate housing is a crucial risk factor and barrier to engagement in HIV care for SCY. The provision of supportive housing for homeless people living with HIV is an effective strategy to improve viral suppression and survival [68, 69, 70]. SCY have the right to safe and adequate housing [25], and innovative approaches to adapt the Housing First for homeless youth model [71] for SCY in Kenya could address this right.

This study has limitations. First, this was a secondary analysis, and our interview guides did not explicitly ask about all the stages of the HIV prevention‐care continuum, and therefore, it is likely that our results are not a comprehensive picture of strategies to improve engagement. Further, the data used in this analysis are 5 years old. However, the findings are likely still highly relevant as the delivery of HIV services to this population during the COVID‐19 pandemic was disrupted and SCY still lack tailored and responsive services to their complex HIV‐related needs across the country. Next, while our study included SCY aged 15–24, it did not include younger adolescents aged 10–14 who may have different perspectives on these issues, nor was the HIV status of participants known. SCY living with HIV would likely have different perspectives and experiences engaging in the HIV prevention‐care continuum and should be engaged in future research to inform the design and development of strategies to improve the delivery of care. Nevertheless, the present study has strengths. This investigation included SCY living across several counties, and it is likely the results reflect SCY's perspectives across these geographic locations. In addition, our study engaged a range of individuals, including healthcare providers, policymakers and community members, which provided a breadth of insight into potential contextually relevant strategies to improve SCY's engagement in the HIV prevention‐care continuum.

## 5 CONCLUSIONS

This study identified contextually relevant strategies for engaging SCY in the HIV prevention‐care continuum that should be adapted, piloted and tested to determine their effectiveness. Notably, this work emphasizes the importance of addressing intersecting structural and social factors across the HIV prevention‐care continuum for SCY. Given the strong associations between homelessness, SGBV, HIV acquisition and poor treatment outcomes, we suggest there is a need to prioritize SGBV prevention and structural interventions, such as cash transfers, safe and secure housing, and food provision for SCY to reduce their risk of acquiring HIV and improve care and treatment outcomes.

## COMPETING INTERESTS

The authors declare no competing interests.

## AUTHORS’ CONTRIBUTIONS

LE, DA and PB conceptualized the study. PS and LE analysed the data. LE drafted the manuscript. LE, PS, EA, DA and PB contributed to the final manuscript.

## FUNDING

Funding for this study was provided through a Canadian Institutes of Health Research (CIHR) Chair of Applied Public Health (Sponsor #0000305843) to PB.

## Data Availability

The data are available upon reasonable request.

## References

[1] UNAIDS . Women and HIV: a spotlight on adolescent girls and young women. Geneva; 2019.

[2] Enane LA , Davies MA , Leroy V , Edmonds A , Apondi E , Adedimeji A , et al. Traversing the cascade: urgent research priorities for implementing the “treat all” strategy for children and adolescents living with HIV in sub‐Saharan Africa. J Virus Erad. 2018;4:40–6. 30515313 10.1016/S2055-6640(20)30344-7 PMC6248846

[3] Green D , Tordoff DM , Kharono B , Akullian A , Bershteyn A , Morrison M , et al. Evidence of sociodemographic heterogeneity across the HIV treatment cascade and progress towards 90‐90‐90 in sub‐Saharan Africa – a systematic review and meta‐analysis. J Int AIDS Soc. 2020;23(3):e25470. 32153117 10.1002/jia2.25470 PMC7062634

[4] Maskew M , Bor J , MacLeod W , Carmona S , Sherman GG , Fox MP . Adolescent HIV treatment in South Africa's national HIV programme: a retrospective cohort study. Lancet HIV. 2019;6(11):e760–e768. 31585836 10.1016/S2352-3018(19)30234-6 PMC7119220

[5] Zanoni BC , Archary M , Buchan S , Katz IT , Haberer JE . Systematic review and meta‐analysis of the adolescent HIV continuum of care in South Africa: the Cresting Wave. BMJ Glob Health. 2016;1(3):e000004. 10.1136/bmjgh-2015-000004 PMC5321340 28588949

[6] Meloni ST , Agaba P , Chang CA , Yiltok E , Oguche S , Ejeliogu E , et al. Longitudinal evaluation of adherence, retention, and transition patterns of adolescents living with HIV in Nigeria. PLoS One. 2020;5(7):e0236801. 10.1371/journal.pone.0236801 PMC7394430 32735566

[7] Enane LA , Vreeman RC , Foster C . Retention and adherence: global challenges for the long‐term care of adolescents and young adults living with HIV. Curr Opin HIV AIDS. 2018;13(3):212–9. 29570471 10.1097/COH.0000000000000459

[8] Haghighat R , Toska E , Bungane N , Cluver L . The HIV care cascade for adolescents initiated on antiretroviral therapy in a health district of South Africa: a retrospective cohort study. BMC Infect Dis. 2021;21(1):60. 33435861 10.1186/s12879-020-05742-9 PMC7805141

[9] UNAIDS . Miles to go: closing gaps, breaking barriers, righting injustices. Global AIDS Update 2018. Geneva; 2018.

[10] Ritchwood TD , Ba A , Ingram LD , Atujuna M , Marcus R , Ntlapo N , et al. Community perspectives of South African adolescents’ experiences seeking treatment at local HIV clinics and how such clinics may influence engagement in the HIV treatment cascade: a qualitative study. AIDS Care. 2020;32(1):83–8. 10.1080/09540121.2019.1653442 PMC6883151 31402674

[11] Enane LA , Apondi E , Omollo M , Toromo JJ , Bakari S , Aluoch J , et al. I just keep quiet about it and act as if everything is alright – the cascade from trauma to disengagement among adolescents living with HIV in western Kenya. J Int AIDS Soc. 2021;24(4):e25695. 33838007 10.1002/jia2.25695 PMC8035676

[12] Bekker LG , Johnson L , Wallace M , Hosek S . Building our youth for the future. J Int AIDS Soc. 2015;18:1–7. 10.7448/IAS.18.2.20027 PMC4344540 25724512

[13] Pettifor A , Nguyen NL , Celum C , Cowan FM , Go V , Hightow‐Weidman L . Tailored combination prevention packages and PrEP for young key populations. J Int AIDS Soc. 2015;18(2 Suppl 1):19434. 25724507 10.7448/IAS.18.2.19434 PMC4344537

[14] Pettifor A , Stoner M , Pike C , Bekker LG . Adolescent lives matter: preventing HIV in adolescents. Curr Opin HIV AIDS. 2018;13(3):265–73. 29528850 10.1097/COH.0000000000000453 PMC5902132

[15] Govender K , Masebo WGB , Cowden RG , Nyamaruze P , Schunter BT , Bains A . HIV prevention in adolescents and young people in the Eastern and Southern African region: a review of key challenges impeding actions for an effective response. Open AIDS J. 2018;12:53–67. 30123385 10.2174/1874613601812010053 PMC6062910

[16] UNAIDS . UNAIDS Terminology Guidelines. 2015.

[17] Bekker LG , Johnson L , Wallace M , Hosek S , Pettifor A , Nguyen NL , et al. HIV and adolescents: focus on young key populations. J Int AIDS Soc. 2015;18(2):20076.

[18] Baggaley R , Armstrong A , Dodd Z , Ngoksin E , Krug A . Young key populations and HIV: a special emphasis and consideration in the new WHO Consolidated Guidelines on HIV Prevention, Diagnosis, Treatment and Care for Key Populations. J Int AIDS Soc. 2015;18(2 Suppl 1):19438. 25724509 10.7448/IAS.18.2.19438 PMC4344541

[19] Wolf RC , Bingham T , Millett G , Wilcher R . Building the evidence base to optimize the impact of key population programming across the HIV cascade. J Int AIDS Soc. 2018;21(Suppl 5):e25146. 30033673 10.1002/jia2.25146 PMC6055132

[20] Braitstein P , Ayuku D , DeLong A , Makori D , Sang E , Tarus C , et al. HIV prevalence in young people and children living on the streets, Kenya. Bull World Health Organ. 2019;97(1):33–41. 30618463 10.2471/BLT.18.210211 PMC6307507

[21] Goldblatt A , Kwena Z , Lahiff M , Agot K , Minnis A , Prata N , et al. Prevalence and correlates of HIV infection among street boys in Kisumu, Kenya. PLoS One. 2015;10(10):e0140005. 26461494 10.1371/journal.pone.0140005 PMC4604137

[22] Braitstein P , DeLong A , Ayuku D , Ott M , Atwoli L , Galárraga O , et al. Association of care environment with HIV incidence and death among orphaned, separated, and street‐connected children and adolescents in western Kenya. JAMA Netw Open. 2021;4(9):e2125365. 10.1001/jamanetworkopen.2021.25365 34529063 PMC8446813

[23] Winston SE , Chirchir AK , Muthoni LN , Ayuku D , Koech J , Nyandiko W , et al. Prevalence of sexually transmitted infections including HIV in street involved adolescents in western Kenya. Sex Transm Infect. 2015;91(5):305. 10.1136/sextrans-2014-051797 PMC4518741 25714102

[24] Shah P , Kibel M , Ayuku D , Lobun R , Ayieko J , Keter A , et al. A pilot study of “peer navigators” to promote uptake of HIV testing, care and treatment among street‐connected children and youth in Eldoret, Kenya. AIDS Behav. 2019;23(4):908–919. 30269232 10.1007/s10461-018-2276-1 PMC6458975

[25] Office of the United Nations High Commissioner for Human Rights . G2017, General comment No. 21 on children in street situations. Geneva; 2017.

[26] Gayapersad A , Embleton L , Shah P , Kiptui R , Ayuku D , Braitstein P . Using a sociological conceptualization of stigma to explore the social processes of stigma and discrimination of children in street situations in western Kenya. Child Abuse Negl. 2020;104803. 10.1016/j.chiabu.2020.104803 33220945 PMC8128938

[27] Embleton L , Shah P , Gayapersad A , Kiptui R , Ayuku D , Wachira J , et al. Exploring patient–provider interactions and the health system's responsiveness to street‐connected children and youth in Kenya: a qualitative study. BMC Health Serv Res. 2021;21(1):363. 33874934 10.1186/s12913-021-06376-6 PMC8056657

[28] Cumber SN , Tsoka‐Gwegweni JM . Characteristics of street children in Cameroon: a situational analysis of demographic, socio‐economic and behavioural profiles and challenges. Afr J Prim Health Care Med. 2016;8(1):e1–9. 10.4102/phcfm.v8i1.1076 PMC5125264 28155316

[29] Kudrati M , Plummer ML , Yousif ND . Children of the sug: a study of the daily lives of street children in Khartoum, Sudan, with intervention recommendations. Child Abus Negl. 2008;32(4):439–48. 10.1016/j.chiabu.2007.07.009 18457872

[30] Arum C , Fraser H , Artenie AA , Bivegete S , Trickey A , Alary M , et al. Homelessness, unstable housing, and risk of HIV and hepatitis C virus acquisition among people who inject drugs: a systematic review and meta‐analysis. Lancet Public Health. 2021;2667(21):1–15. 10.1016/S2468-2667(21)00013-X PMC8097637 33780656

[31] Ayaya S , DeLong A , Embleton L , Ayuku D , Sang E , Hogan J , et al. Prevalence, incidence and chronicity of child abuse among orphaned, separated, and street‐connected children and adolescents in western Kenya: what is the impact of care environment? Child Abus Negl. 2021;104920. 10.1016/j.chiabu.2020.104920 PMC8289926 33485648

[32] Hills F , Meyer‐Weitz A , Asante KO . The lived experiences of street children in Durban, South Africa: violence, substance use, and resilience. Int J Qual Stud Health Well‐being. 2016;11:30302 10.3402/qhw.v11.30302 27291160 PMC4904070

[33] Embleton L , Shah P , Gayapersad A , Kiptui R , Ayuku D , Braitstein P . Characterizing street‐connected children and youths’ social and health inequities in Kenya: a qualitative study. Int J Equity Health. 2020;19(1):147. 32859193 10.1186/s12939-020-01255-8 PMC7455900

[34] Sorber R , Winston S , Koech J , Ayuku D , Hu L , Hogan J , et al. Social and economic characteristics of street youth by gender and level of street involvement in Eldoret, Kenya. PLoS One. 2014;9(5):e97587. 24827584 10.1371/journal.pone.0097587 PMC4020866

[35] Human Rights Watch . “Where do you want us to go?”: abuses against street children in Uganda. 2014.

[36] Mandalazi P , Banda C , Umar E . Street children's vulnerability to HIV and sexually transmitted infections in Malawian cities. Malawi Med J. 2013;25(1):1–4. 23717747 PMC3653190

[37] Mudingayi A , Lutala P , Mupenda B . HIV knowledge and sexual risk behavior among street adolescents in rehabilitation centres in Kinshasa; DRC: gender differences. Pan Afr Med J. 2011;10:1–17. 10.4314/pamj.v10i0.72233 PMC3224059 22187605

[38] Embleton L , Wachira J , Kamanda A , Naanyu V , Winston S , Ayuku D , et al. “Once you join the streets you will have to do it”: sexual practices of street children and youth in Uasin Gishu County, Kenya. Reprod Health. 2015;12:1–11. 26573581 10.1186/s12978-015-0090-z PMC4647324

[39] Embleton L , Wachira J , Kamanda A , Naanyu V , Ayuku D , Braitstein P . Eating sweets without the wrapper: perceptions of HIV and sexually transmitted infections among street youth in western Kenya. Cult Health Sex. 2016;18(3):337–48. 26394208 10.1080/13691058.2015.1082626 PMC4854983

[40] Olaleye AO , Obiyan MO , Folayan MO . Factors associated with sexual and reproductive health behaviour of street‐involved young people: findings from a baseline survey in Southwest Nigeria. Reprod Health. 2020;17(1):94. 32527331 10.1186/s12978-020-00937-4 PMC7291518

[41] Save the Children . The chronic urban emergency in Rift Valley Kenya: report from Profiling Children Connected to the Streets in Rift Valley Province. London: Save the Children/UNICEF; 2012.

[42] Kaime‐Atterhög W , Ahlberg BM . Are street children beyond rehabilitation? Understanding the life situation of street boys through ethnographic methods in Nakuru, Kenya. Child Youth Serv Rev. 2008;30:1345–54.

[43] Railway Children . Children on the streets of Kitale: headcount findings 2015. 2015.

[44] Republic of Kenya Ministry of Health . Kenya HIV Estimates: Report 2018. Nairobi; 2018.

[45] Embleton L , Ayuku D , Makori D , Kamanda A , Braitstein P . Causes of death among street‐connected children and youth in Eldoret, Kenya. BMC Int Health Hum Rights. 2018;18:19 10.1186/s12914-018-0160-8 29764412 PMC5952842

[46] Kibel M , Pierzchalski J , Gorfinkel L , Embleton L , Ayuku D , Hogg R , et al. Standardized mortality ratio between street‐connected youth and the general youth population in an urban setting in sub‐Saharan Africa. Glob Health Action. 2020;13(1):1802097. 32819217 10.1080/16549716.2020.1802097 PMC7480584

[47] Kibel M , Shah P , Ayuku D , Makori D , Kamaara E , Choge E , et al. Acceptability of a pilot intervention of voluntary medical male circumcision and HIV education for street‐connected youth in western Kenya. J Adolesc Health. 2019;64(1):43–8. 30327277 10.1016/j.jadohealth.2018.07.027

[48] Embleton L , Di Ruggiero E , Logie CH , Ayuku D , Braitstein P . Piloting an evidence‐based intervention for HIV prevention among street youth in Eldoret, Kenya. Int J Public Health. 2020;64(4):433–43. 10.1007/s00038-020-01349-8 PMC7275002 32270232

[49] Embleton L , Di Ruggiero E , Logie C , Ayuku D , Braitstein P . Improving livelihoods and gender equitable attitudes of street‐connected young people in Eldoret, Kenya: results from a pilot evidence‐based intervention. Health Soc Care Community. 2020;29(1):227–240. 32633059 10.1111/hsc.13086

[50] Embleton L , Di Ruggiero E , Odep Okal E , Chan A , Logie C , Ayuku D , et al. Adapting an evidence‐based gender, livelihoods, and HIV prevention intervention with street‐connected young people in Eldoret, Kenya. Glob Public Health. 2019;14(12):1703–17. 31162989 10.1080/17441692.2019.1625940 PMC6906550

[51] Kibel M , Nyambura M , Embleton L , Kiptui R , Galarraga O , Apondi E , et al. Enabling Adherence to Treatment (EAT): a pilot study of a combination intervention including modified directly observed therapy to improve HIV treatment outcomes among street‐connected individuals in western Kenya. 2021. 10.1186/s12913-023-10215-1 PMC10691070 38037045

[52] Einterz RM , Kimaiyo S , Mengech HNK , Khwa‐Otsyula BO , Esamai F , Quigley F , et al. Responding to the HIV pandemic: the power of an academic medical partnership. Acad Med. 2007;82:812–8. 17762264 10.1097/ACM.0b013e3180cc29f1

[53] Embleton L , Ott MA , Wachira J , Naanyu V , Kamanda A , Makori D , et al. Adapting ethical guidelines for adolescent health research to street‐connected children and youth in low‐ and middle‐income countries: a case study from western Kenya. BMC Med Ethics. 2015;16:89 10.1186/s12910-015-0084-y 26687378 PMC4684915

[54] Creswell JW . Qualitative inquiry and research design: choosing among five traditions. 2nd ed. Thousand Oaks, CA: Sage Publications; 2007.

[55] Braun V , Clarke V . Using thematic analysis in psychology. Qual Res Psychol. 2006;3:77–101.

[56] Winston SE , Chirchir AK , Muthoni LN , Ayuku D , Koech J , Nyandiko W , et al. Prevalence of sexually transmitted infections including HIV in street‐connected adolescents in western Kenya. Sex Transm Infect. 2015;91(5):353–9. 25714102 10.1136/sextrans-2014-051797 PMC4518741

[57] Wachira J , Kamanda A , Embleton L , Naanyu V , Ayuku D , Braitstein P . “Pregnancy has its advantages”: the voices of street connected children and youth in Eldoret, Kenya. PLoS One. 2016;11(3):e0150814. 26942724 10.1371/journal.pone.0150814 PMC4778759

[58] Wachira J , Kamanda A , Embleton L , Naanyu V , Winston S , Ayuku D , et al. Initiation to street life: a qualitative examination of the physical, social, and psychological practices in becoming an accepted member of the street youth community in western Kenya. BMC Public Health. 2015;15(1):569. 26087662 10.1186/s12889-015-1942-8 PMC4473841

[59] Kibel M , Pierzchalski J , Gorfinkel L , Embleton L , Ayuku D , Hogg R , et al. Standardized mortality ratios between street‐connected young people and the general age‐equivalent population in an urban setting in Kenya from 2010 to 2015. Glob Health Action. 2020;13(1):1802097. 32819217 10.1080/16549716.2020.1802097 PMC7480584

[60] World Health Organization . Consolidated guidelines on HIV prevention, diagnosis, treatment and care for key populations –2016 update. World Health Organization; 2016.

[61] FHI 360, LINKAGES . Key Population Program Implementation Guide. Washington, DC; 2016.

[62] Medley A , Kennedy C , O'Reilly K , Sweat M . Effectiveness of peer education interventions for HIV prevention in developing countries: a systematic review and meta‐analysis. AIDS Educ Prev. 2009;21(3):181–206. 19519235 10.1521/aeap.2009.21.3.181 PMC3927325

[63] Chandra‐Mouli V , Lane C , Wong S . What does not work in adolescent sexual and reproductive health: a review of evidence on interventions commonly accepted as best practices. Glob Health Sci Pract. 2015;3(3):333–40. 26374795 10.9745/GHSP-D-15-00126 PMC4570008

[64] Maticka‐Tyndale E , Barnett JP . Peer‐led interventions to reduce HIV risk of youth: a review. Eval Program Plann. 2010;33(2):98–112. 19647874 10.1016/j.evalprogplan.2009.07.001

[65] UNAIDS . The youth bulge and HIV: UNAIDS explainer. Geneva; 2018.

[66] Olaleye AO , Obiyan MO , Folayan MO . Factors associated with sexual and reproductive health behaviour of street‐involved young people: findings from a baseline survey in Southwest Nigeria. Reprod Health. 2020;17(1):94 10.1186/s12978-020-00937-4 32527331 PMC7291518

[67] Richterman A , Thirumurthy H . The effects of cash transfer programmes on HIV‐related outcomes in 42 countries from 1996 to 2019. Nat Hum Behav. 2022 10.1038/s41562-022-01414-7 35851840

[68] Peng Y , Hahn RA , Finnie RKC , Cobb J , Williams SP , Fielding JE , et al. Permanent supportive housing with housing first to reduce homelessness and promote health among homeless populations with disability: a community guide systematic review. J Public Health Manag Pract. 2020;26:404–41. 32732712 10.1097/PHH.0000000000001219 PMC8513528

[69] Schwarcz SK , Hsu LC , Vittinghoff E , Vu A , Bamberger JD , Katz MH . Impact of housing on the survival of persons with AIDS. BMC Public Health. 2009;9:220. 19583862 10.1186/1471-2458-9-220 PMC2728715

[70] Buchanan D , Kee R , Sadowski LS , Garcia D . The health impact of supportive housing for HIV‐positive homeless patients: a randomized controlled trial. Am J Public Health. 2009;9(Suppl 3):S675–80. 10.2105/AJPH.2008.137810 PMC2774195 19372524

[71] Kozloff N , Adair CE , Palma Lazgare LI , Poremski D , Cheung AH , Sandu R , et al. “Housing first” for homeless youth with mental illness. Pediatrics. 2016;138(4):e20161514. 27681009 10.1542/peds.2016-1514

